# The know-do gap in quality of health for chronic non-communicable diseases in rural China

**DOI:** 10.3389/fpubh.2022.953881

**Published:** 2022-08-18

**Authors:** Sha Meng, Qingzhi Wang, Yuju Wu, Hao Xue, Linhua Li, Ruixue Ye, Yunwei Chen, Lucy Pappas, Muizz Akhtar, Sarah-Eve Dill, Sean Sylvia, Huan Zhou, Scott Rozelle

**Affiliations:** ^1^Department of Health Behavior and Social Medicine, West China School of Public Health, West China Fourth Hospital, West China Hospital, Sichuan University, Chengdu, China; ^2^Department of Operation Management, West China Hospital, Sichuan University, Chengdu, China; ^3^Stanford Center on China's Economy and Institutions, Freeman Spogli Institute for International Studies, Stanford University, Stanford, CA, United States; ^4^Department of Health Policy and Management, Gillings School of Global Public Health, University of North Carolina at Chapel Hill, Chapel Hill, NC, United States

**Keywords:** know-do gap, provider quality, standardized patients, diabetes, angina, rural health system

## Abstract

Proper management of non-communicable diseases (NCDs) is a severe challenge to China's rural health system. This study investigates what influences the poor medical treatment of NCDs (diabetes and angina) by evaluating the “know-do gap” between provider knowledge and practice. To determine whether low levels of provider knowledge low quality of patient care is the primary constraint on the quality of NCDs diagnosis and treatment in rural China. Providers from Village Clinics (VC) and Township Health Centers (THC), and Standardized Patients (SP) were selected by a multi-stage random sampling method. Clinical vignettes were administered to 306 providers from 103 VCs and 50 THCs in rural Sichuan Province. SPs presented diabetes symptoms completed 97 interactions with providers in 46 VCs and 51 THCs; SPs presented angina symptoms completed 100 interactions with providers in 50 VCs and 50 THCs. Process quality, diagnosis quality, and treatment quality were assessed against national standards for diabetes and angina. Two-tailed *T*-tests and tests of proportions for continuous outcomes and tests of proportions for binary dependent variables were used to compare vignette and SP results. Differences between vignette and SP data calculated the know-do gap. Regression analyses were used to examine the providers/facility characteristics and knowledge/practice associations. THC providers demonstrated significantly more knowledge in vignettes and better practices in SP visits than VC providers. However, levels of knowledge were low overall: 48.2% of THC providers and 28.2% of VC providers properly diagnosed type 2 diabetes, while 23.8% of THC providers and 14.7% of VC providers properly diagnosed angina. With SPs, 2.1% of THC providers and 6.8% of VC providers correctly diagnosed type 2 diabetes; 25.5% of THC providers and 12.8% of VC providers correctly diagnosed angina. There were significant know-do gaps in diagnosis process quality, diagnosis quality, and treatment quality for diabetes (*p* < 0.01), and in diagnosis process quality (*p* < 0.05) and treatment quality for angina (*p* < 0.01). Providers in rural China display low levels of knowledge when treating diabetes and angina. Despite low knowledge, evidence of the know-do gap indicates that low-quality healthcare is the primary constraint on the quality of NCD diagnosis and treatment in rural China. Our research findings provide a new perspective for the evaluation of the medical quality and a technical basis for the development of new standardized cases in the future.

## Introduction

Chronic non-communicable diseases (NCDs) are a large and growing public health problem worldwide (1, 2). In fact, NCDs, which are often misdiagnosed or untreated (3), are estimated to account for over half of all global deaths (1). Furthermore, projections indicate that the global impact of common NCDs, including diabetes mellitus (hereafter, diabetes) and cardiovascular diseases, are likely to continue increasing (4). A study published by the International Diabetes Federation (IDF) found that as of 2019, approximately 463 million adults between the ages of 20 and 79 years had diabetes; 4.2 million people died from diabetes complications in the same year (5). Additionally, common cardiovascular diseases, such as angina and other heart conditions, affect more than 1.26 billion people, globally, and were the leading causes of premature deaths in 2017 (6).

Reducing the prevalence of diabetes and cardiovascular diseases in rural communities in China is a crucial step toward lowering the total disease burden of NCDs. In China, large percentages of the population, particularly those living in rural communities, are affected by NCDs, including diabetes and angina. China is ranked number one globally for diabetes cases at 116 million people (5). Moreover, the highest rates of diabetes in China are found in rural areas with diabetes affecting 14.8% of all rural residents, which is significantly higher than the national average of 10.9% (7). Like diabetes, common cardiovascular diseases, notably angina, account for an alarmingly high rate of deaths in China, with rural residents also disproportionally affected (8). In 2016, the prevalence of angina among rural residents was 309 per 100,000, which was higher than in urban areas, which reported 265 per 100,000 (9).

Why are NCDs so prevalent in rural communities in China? One possible explanation is poor medical treatment. According to the literature on this subject, subpar clinical quality and diagnostic inaccuracies persist in Primary Health Care (PHC) centers in rural China, which are in part responsible for managing illnesses, including NCDs (10). Township Health Centers (THCs) and Village Clinics (VCs) are two examples of PHC centers in rural China, both of which are in the bottom two tiers of the rural health system (11). While few studies have focused specifically on rates of NCD misdiagnosis, one study conducted in rural communities across three provinces (Sichuan, Shaanxi, and Anhui) reported significantly high rates of misdiagnosis (12). Using incognito Standardized Patients (SPs)—individuals from the local community trained to act as real patients with a set of simulated symptoms or problems—the research team found that the sampled providers in the THCs correctly diagnosed tuberculosis only 38% of the time; providers in the sample's VCs only correctly diagnosed tuberculosis 28% of the time (12). Another study conducted in rural areas of southern Shaanxi province found that when a sample of incognito SPs presented symptoms of dysentery or unstable angina, village doctors did not ask the recommended questions used to assess and evaluate a patient's condition 82% of the time and only correctly diagnosed the patient's condition 26% of the time (11). Moreover, a 2013 nationally representative survey in China found a total diabetes prevalence of 11%, with more than 60% of affected individuals unaware they had diabetes (7). In 2019, 231.9 million (50.1%) of the 463 million people worldwide living with did not know they had diabetes (5). A nationally representative survey in 2013 involving 170,287 participants found that the prevalence of diabetes in China was 10.9%, and the proportion of undiagnosed diabetes patients was 63% (13). Of those with diabetes, only 37 % were aware of their condition and only 32% were receiving treatment (7). However, the prevalence of diabetes in the United States (10.8%) was similar to that in China (10.9%) from 2011 to 2014, but only about 1.2% of cases went undiagnosed (14). In rural areas specifically, 71% of the sampled individuals with diabetes were unaware that they were diabetic, while only 25% of the sampled individuals were receiving treatment for their condition (7). These studies indicate that there is a serious deficiency in the diagnostic capabilities of rural medical providers.

One commonly cited source of poor medical treatment is the “know-do gap.” The know-do gap is the discrepancy between a provider's diagnostic knowledge or education (the “know”), and their actual performance when consulting a patient (the “do”). In developing settings, previous research has identified significant know-do gaps among physicians in India (13–16), Tanzania (17), and China (12), for example. In China's rural health system, where provider knowledge is already dangerously low (11, 12, 18, 19), the possibility of substantial know-do gaps presents a concerning threat to the successful treatment of common NCDs. Therefore, understanding this gap is critical to understanding the root causes for poor health care quality and for informing improvement efforts in public health systems; however, to our knowledge, no studies have explicitly focused on how the know-do gap influences NCD diagnosis and treatment in rural China.

Few studies focus on the early diagnosis and management of diseases, especially for NCDs (10), with the main reason is being that quality is difficult to measure. Current methods mainly measure the performance degree of basic medical functions perceived by patients through scale (PCAT scales, SERVQUAL tool), the Technique for Order of Preference by Similarity to Ideal Solution (TOPSIS), and observations to evaluate the service quality of primary medical institutions (20–22). All of the methods are affected by the Hawthorne Effect, thus introducing bias (21). However, the clinical vignette method and the standardized patient (SP) method, which have been gradually introduced to research around the world, are considered the gold standards for measuring quality of care in healthcare settings (23). Both methods are becoming increasingly popular in low- and middle-income countries, thus creating new opportunities to answer multiple research questions about quality of care.

To better understand the know-do gap and the quality of health care in rural China, and more specifically, whether the know-do gap exists and how large it is in rural China, our study has three objectives. First, we assess whether rural THC and VC providers possess or lack knowledge on diagnosing diabetes and angina (the “know”). To achieve this goal, we assess provider knowledge through the clinical vignette method, which involves collecting data by presenting hypothetical situations to health care providers and asking a set of directed questions to reveal their knowledge on how to diagnose and treat the diseases. Second, using the SP method, we measure the performance of THC and VC providers when dealing with “actual” patients. Examining the results of the SP visits, we determine whether the providers, in practice, succeed or fail to properly diagnose/treat/manage diabetes and angina. Finally, we compare the clinical vignette results (the “know”) with SP results (the “do”) to identify the gap between provider knowledge and practice, thereby determining whether it is low levels of provider knowledge or low quality of patient care that is the primary constraint on the quality of NCD diagnosis and treatment in rural China. Our research on the diagnosis and treatment quality of diabetes and angina provides a scientific reference for the study on clinical care quality of NCDs, provides technical support for the practice of new developing standardized cases of NCDs, and provides innovative ideas and directions for future research on clinical care quality.

## Methods

### Ethical approval

Approval was obtained from the Institutional Review Board (IRB) of Sichuan University, China (protocol number: K2019021). Informed consent was obtained verbally from all providers participating in the study. The IRB approved the procedure whereby providers consented to SP visits “at some point in the next 6 months.” Consent from THC and VC providers was obtained as part of the facility survey ~4 weeks before SP visits. All individuals who participated as SPs were trained to protect themselves from any invasive tests or procedures.

### Sample and participants

The data used in this study were collected from several rural counties in Sichuan Province in western China. Sichuan is a relatively rural province with 47.7% of residents living in rural areas (18, 24). The per capita income of rural residents in the sample city was RMB 14,380 (United States Dollar or USD 2,157) in 2019 which was slightly less than the median per capita income for rural areas in China in the same year (RMB 14,617; USD 2,253) (18, 24). Regarding the ethnicity of the sample, Han individuals accounted for the majority of all five sampled counties (93.89%) which is a similar ethnic distribution of Han individuals nationwide (91.59%).

Data on rural health facilities were collected during 2019 in rural Sichuan Province using a multistage cluster sampling approach (25, 26). First, five rural counties in the province were randomly selected. To select THCs, ten townships were randomly chosen from each sampled county. Townships that housed the county seat (which are typically more urbanized) were excluded. In total, 50 townships were randomly chosen, and all THCs within each sampled township were enrolled in the study, totaling 50 THCs. To select VCs, the research team first randomly selected two villages from each selected township. In selecting villages, we only selected villages with populations of 800 or more people, due to the increased likelihood that villages with smaller populations would not have a VC in the village. In total, 100 villages were selected, and within each selected village, all VCs were surveyed, totaling 103 VCs. Thus, our final sample included 50 THCs and 103 VCs.

### Provider and facility surveys

Trained enumerators administered both provider and facility surveys in the selected THCs and VCs. The provider survey collected information on the demographic characteristics of the health care providers, including provider age, gender, years of experience, highest level of educational attainment overall, highest level of educational attainment in the medical field/medical qualifications, length of career (years), monthly salary (Renminbi or RMB), number of patients seen weekly, number of hours worked weekly, and the average duration of a consultation (minutes). The facility survey documented the medical equipment available at the health care facility (both THCs and VCs) as well as information on the population in the health facility catchment areas, the patient volume of facilities, the number of providers working full time, and the distance between each facility and the county hospital.

### Clinical vignettes

Clinical vignettes were administered by trained enumerators to assess provider knowledge regarding diagnosis and treatment of patients. Enumerators acted as “mock patients” and were trained to use scripted responses to provider questions and exam requests. All enumerators spent several days practicing their scripts in groups of two to ensure that the clinical vignettes were implemented in a standardized way. Enumerators were then dispatched to each sampled county by the survey team. During the survey, two enumerators presented the vignette to each provider, in either THCs or VCs. One enumerator assumed the role of the “mock patient” and the other took the role of the “facilitator.” The facilitator gave instructions to the provider and documented the interaction.

To begin the diabetes vignette, the facilitator informed the provider that either a 52-year-old male patient or a 49-year-old female patient was visiting the clinic. For the angina vignette, the provider was informed by the facilitator to expect a 45-year-old male patient. The mock patient then used a standardized opening statement to tell the provider about their symptoms. If the mock patient was presenting symptoms of diabetes, they would open with the statement, “Doctor, I've been very thirsty recently.” If the mock patient was presenting symptoms of angina, they would open with the statement, “Doctor, I've had chest pains recently.” Providers were then asked to proceed as they would with a real patient and were told that the mock patient would answer any questions asked and comply with any instructions given. During the interaction, the facilitator documented the questions of the providers, diagnostic examinations requested, stated diagnosis, treatment plans, and whether the providers referred the patient to another provider.

### Standardized patients

To further assess provider performance, the quality of provider diagnoses, and prescribed treatments, our study used incognito standardized patients (SPs), which are regarded as an international gold standard for measuring the quality of medical care (27). The use of SPs has been validated in multiple studies in China (18, 19, 28, 29). In our study, each SP was trained to present symptoms of diabetes or angina. The use of SPs to present symptoms of diabetes and angina was appropriate for our study approach as there are no obvious or outward physiological symptoms of either condition. Because of this, the likelihood that SPs would be exposed to invasive procedures or tests was low. To our knowledge, this is the first study to examine both angina and diabetes care using the SP approach in rural China.

SPs were trained to follow scripts for each disease. Each script included disease symptoms as well as a detailed background story for each case. The SP script for diabetes was developed with the help of providers from the Western China Hospital of Sichuan University. Our script for angina was developed using the Das et al. and Mohanan et al. (15, 16) study of angina in rural India and a study of angina and tuberculosis in rural China (12, 16).

SP data were collected 1 month after clinical vignettes were completed. An initial group of 15 SP volunteers between the ages of 40 and 50 years was recruited based on (a) a familiarity with the local dialect; (b) having an educational background similar to the SP scripted background story; (c) having a career relevant to the SP scripted background story; (d) not having friends and relatives working in the sample THCs or VCs. Additionally, volunteers underwent physical examinations to ensure they had no obvious symptoms of other diseases that would interfere with the diagnosis and treatment of the health care providers. After the physical exams had been completed, ten SPs were selected from the volunteer pool (eight male, two female) and asked to act as patients with diabetes or angina. These volunteers received additional training over a 2-week period in order to give consistent presentations to providers in the sample health facilities.

SP case scripts were designed on the basis of international and Chinese diagnostic criteria, and the scripts used were the same in all the Clinical vignettes. SP scripts include descriptions of symptoms, detailed backstories, and standardized answers to questions doctor might ask. Local residents were recruited to act as SPs who met the required age for each case (for diabetes, SPs were 45–50 years old, for angina, SPs were 35–45 years old). Volunteers were trained to present the cases written in the script to the sample providers. Additionally, volunteers underwent physical examinations to ensure they had no obvious symptoms of other diseases that would interfere with the diagnosis and treatment of the health care providers. After the physical exams had been completed, ten SPs were selected from the volunteer pool (eight male, two female) and asked to act as patients with diabetes or angina. These volunteers received additional training over a 2-week period in order to give consistent presentations to providers in the sample health facilities.

Two pre-tests were conducted for both diabetes and angina cases to ensure that the designed case scripts could fully answer any questions that doctors might ask, and to continuously improve the standardized code of conduct for patients, including the corresponding plan designed to avoid invasive examination. The pretests also served as time to establish the answer principles for the questions not yet established in the cases. Finally, the symptoms and background stories of the two cases were confirmed to be consistent with the reality of residents in rural areas of China. SPs could clearly describe the clinical symptoms of the case, and could answer all questions raised by the investigated doctors in a standardized and accurate manner.

The training process for standardized patients included three stages: script training, indoor simulation exercise, and field pre-investigation. During script training, SPs were instructed how to accurately remember their patient symptoms and story background, how to answer questions according to the basic principle of non-disclosure staying consistent with the characteristics of the case, and how to use the recording equipment to collect clinic process data. The investigators led and participated in the SP training process.

Before the SPs entered the medical institution, the investigator helped the SP practice the script, answer the principle, visit the procedure principle, and become familiar with the operation of the recording equipment again. At the end of the visit, the standardized patient and the investigator met and immediately filled in the visit recall questionnaire. Meanwhile, the investigator needed to confirm whether the whole visit process was wrong according to the recording content. If any problem is found, the standardized patient is required to return to the medical institution immediately and correct the problem in time according to the principle. Finally, the researcher will confirm that the whole process is successfully completed and correct before leaving the site.

### Know-do gap

Based on the results from the clinical surveys, vignettes, and SP visits, this study aimed to assess the know-do gap in rural China. The know-do gap—discrepancies between what providers have knowledge of, for example, through education and experience, and what they actually do in practice in a clinic during a visit by a “real” patient—is well documented in other contexts (16, 17, 30, 31). The results of know-do gap analysis can help inform policy makers whether interventions targeting knowledge deficits are likely to be effective means of improving the quality of care. Know-do gap analyses can also identify overall poor-quality and search for the underlying reasons (e.g., is it due to poor knowledge; poor incentives; or both).

In our study, the difference between clinical vignettes and SP assessments was used to compare health care provider knowledge and their actual performance. Diagnoses and treatments were deemed correct or incorrect based on predetermined standards. The clinical vignettes collected by enumerators determined the knowledge of providers by testing the accuracy of their diagnoses and treatments for mock patients presenting symptoms of angina and diabetes. The research team then compared these responses with data from SP-based assessments that described what practitioners actually did in practice when they were presented with the same case by incognito SPs. The difference between the two measures is the “know-do gap.”

### Quality measures

This study used three domains to evaluate health care provider performance, which included diagnostic process quality, diagnosis quality, and treatment prescribed. First, we assessed diagnostic process quality. Diagnostic process quality refers to the process in which the provider collects clinical data, including patient history, symptoms, laboratory tests, and auxiliary examination results, by inquiring about a patient's disease symptoms. This serves as the basis and key for diagnosing a particular disease. Diagnostic process quality was determined through diabetes and angina checklists that the study team created by comparing both diagnostic questions that providers asked and medical tests that providers recommended to the checklist of recommended questions/tests outlined in China's national practice guidelines (13). The diabetes and angina checklists (created by this study's team) each consisted of (a) 19 and 15 recommended questions that health care providers should ask patients regarding their symptoms that can help the doctor diagnose diabetes and angina, respectively; (b) 5 and 7 essential questions for diabetes and angina cases, respectively, from all recommended questions that the research team, through adhering to international and Chinese diagnostic standards, deemed to be the most critical information to ask patients when diagnosing a disease; (c) 10 and 6 recommended medical tests for diabetes and angina cases, respectively, that health care providers were expected to recommend for patients; and (d) 4 and 2 essential exam items for diabetes and angina cases, respectively, that health care providers were expected to recommend for patients.

Second, the quality of diagnoses was assessed based on predetermined standards of correctness. Diagnoses were classified as “correct,” “partially correct,” or “incorrect,” based on case management checklists that were written by the research team based on the national practice guidelines for diabetes and angina case management (13). To ensure that diagnoses were given for each interaction, SPs and enumerators were instructed to ask clinicians directly at the conclusion of the visit if a diagnosis had not already been volunteered.

Finally, the quality of treatment from providers, which includes general case management and prescription of medication for alleviating or curing a patient's disease, was assessed. Treatment plans were assessed based on whether the provider prescribed the appropriate medication, gave effective guidance (e.g., on monitoring blood sugar for diabetes, lifestyle adjustments) and accurate referral suggestions, and whether the treatment plan was fundamentally “correct” for the particular case. Treatments were judged to be “correct” or “incorrect” against a predetermined definition of appropriate treatments. Similarly, medication treatments were deemed “correct” if clinicians dispensed any one of the “correct” medications.

### Statistical methods

To meet our first and second objectives, we use data from the clinical vignettes to assess provider knowledge, and data from SP visits to assess provider performance. For each facility, we calculate the mean of correct diagnoses, correct treatment, prescription of appropriate medication, and the number of recommended and essential checklist items completed, using data from clinical vignettes. Next, we use the same methods to analyze the process quality, diagnostic quality, and treatment quality of sampled healthcare providers using data from SP visits. To assess the difference between clinical vignette and SP visits at the township and village levels, we use two-tailed *T*-tests and tests of proportions (χ^2^ tests) for continuous outcomes and tests of proportions (χ^2^ tests) for binary dependent variables.

To meet our final objective, we compare the clinical vignette surveys with the SP surveys to determine if the sample THC and VC providers show significant know-do gaps. The know-do gap is calculated by comparing practitioner answers to clinical vignettes and diagnostic questions and treatment recommendations posed to SPs. Then, we use regression analyses to examine associations between the observable characteristics of providers/practices and their knowledge and practice. We estimate multivariable regressions in which the outcome is provider performance measured as the percentage of diagnostic questions asked for diabetes and angina. We use a fixed-effect model in our analysis to eliminate the effects of county and SP characteristic differences (age, gender, education, working years, and medical certification). We then conduct logistic regressions for the correct diagnosis, correct drug therapy, prescription of potentially harmful treatments, and correct treatments for each case. All regressions control for age, medical qualification, practitioner work hours, and patient volume. Analyses were done using Stata V.15.0 (StataCorp, College Station, TX, USA).

## Results

### Description of providers and facilities

Descriptive statistics of the whole sample in this study are reported in Table 1. Regarding provider characteristics, the majority of providers (64%) worked in a THC (*n* = 196); the remainder (36%) worked in VCs (*n* = 110). The majority of providers were male (62.4%), and their average age was 45 years (Standard Deviation, SD = 8.4). Overall, primary care providers had low levels of education and low levels of formal medical qualification. Specifically, only 14.4% had a bachelor's degree, and the highest level of education completed by the largest share of providers (46.7%) was part of (that is, they did not graduate from high school) or less than high school. Across the whole sample, 43.1% of providers held a “Practicing Physician Certificate” (which is the most advanced degree of China's more general Practicing Physician Certificate, or PPC), while 26.8% had an “Assistant Physician Certificate,” and 29.4% had the most basic certificate required to practice medicine in rural areas: the “Rural Physician Certificate.” In terms of provider experience, on average, providers had worked for 22 years (SD = 9.14). Overall, the average monthly salary was RMB 3670 (SD = 1.45).

**Table 1:** Medical provider and facility characteristics.

**Provider characteristics**	**Total (*****N*** = **306)**		**VC (*****n*** = **110)**		**THC (*****n*** = **196)**		***P***
	***n*/$\bar{x}$**	**%/SD**	***n*/$\bar{x}$**	**%/SD**	***n*/$\bar{x}$**	**%/SD**	
Gender (male)	191	62.42	76	69.09	115	58.67	0.071
Age (years)	44.70	8.44	47.95	6.97	42.86	8.65	<0.01
**Education level**							
Part of or below high school	143	46.73	92	83.64	51	26.02	<0.01
Completed high school	119	38.89	17	15.45	102	52.04	
Completed bachelor's degree	44	14.38	1	0.91	43	21.94	
**PPC**							
Rural physician	90	29.41	80	72.73	10	5.1	<0.01
Assistant physician	82	26.80	21	19.09	61	31.12	
Practicing physician	132	43.14	7	6.36	125	63.78	
Working years	22.3	9.14	25.33	7.90	20.60	9.37	<0.01
Monthly salary (RMB)	3670	1450	2410	950	4370	1180	<0.01
No. of patients weekly	43.49	49.24	35.72	39.15	47.85	53.69	<0.05
Weekly working hours	40.12	27.54	10.73	4.64	56.60	20.34	<0.01
Consulting duration (minutes)	14.18	8.53	17.36	8.94	12.38	7.76	<0.01
**Facility characteristics**	**Total (*****N*** = **153)**		**VCs (*****n*** = **103)**		**THC (*****n*** = **50)**		***P***
	***n*** **/** $\bar{x}$	**%/SD**	***n*** **/** $\bar{x}$	**%/SD**	***n*** **/** $\bar{x}$	**%/SD**	
Glucometer (1 = yes)	125	81.70	75	72.82	50	100	<0.01
Height-weighting scale (1 = yes)	142	92.81	93	90.29	49	98.00	0.083
Electrocardiograph (EKG) (1 = yes)	50	32.68	0	0.00	50	100	<0.01
No. of population in catchment area (thousand)	13.52	23.18	1.77	91.21	37.74	5.76	<0.01
No. of patients in 2018 (thousand)	8.8	1.17	1.45	0.18	24.10	2.41	<0.01
No. of physicians working full time	4.76	0.60	1.18	0.04	12.14	1.32	<0.01
Distance of county hospital (km)	23.20	1.14	23.74	1.41	22.08	1.92	0.494

VC, Village Clinic; THC, Township Health Center; PPC, Practicing Physician Certificate.

Data source: Authors' survey.

Table 1 also presents the descriptive statistics on all facilities (*N* = 153). The survey data have shown that, overall, 82% of the facilities had a glucometer, which was necessary for the recommended exams on the clinical checklist for diabetes. Among the whole sample, 92.8% of facilities had a height/weight scale, while 32% of all facilities had an electrocardiogram (EKG) recording device. Across the THC facilities (*n* = 50) and VC facilities (*n* = 103), all EKG devices were located in THC facilities, and none in any VC facility. Due to the low rate of EKG ownership in VCs, the EKG item on the survey checklist was scored based on whether providers in VCs that did not have an EKG referred patients to another medical institution that possessed an EKG device. In 2018, the average number of patients among all facilities was 8,800.

### Clinical vignette surveys

Tables 2, 3 report results of the clinical vignette surveys for the diabetes and angina cases, respectively, which were both measured by the quality of the provider's process, the quality of the diagnosis, and the quality of the treatment.

**Table 2:** Main outcomes of interactions in clinical vignettes for diabetes cases.

**Main outcomes of interactions in clinical vignettes**	**Total (*N* = 306)**	**VC (*n* = 110)**	**THC (*n* = 196)**	***P***
	**%/*n***	**%/*n***	**%/*n***	
**Process quality**				
Number of recommended questions and exams (out of 29)	6.73	4.83	7.80	<0.01
% of recommended questions and exams	23.20	16.65	26.88	<0.01
Number of essential questions and exams (out of 9)	4.02	3.04	4.56	<0.01
% of essential questions and exams	44.63	33.74	50.62	<0.01
**Diagnosis quality (%)**				
Gave any diagnosis	99.67	100	99.49	0.453
Correct diabetes diagnosis	93.77	87.27	97.44	<0.01
Correct type 2 diabetes diagnosis	40.98	28.18	48.21	<0.01
**Quality of treatment (%)**				
Gave any treatment plan	99.35	99.09	99.49	0.678
Correct treatment	38.82	24.77	46.67	<0.01
Partly correct treatment	89.80	77.06	96.92	<0.01
Gave advice on diet, lifestyle, or exercise	64.14	46.79	73.85	<0.01
Instructed patient to monitor blood glucose regularly	59.21	44.04	67.69	<0.01
Gave any medication	80.07	77.27	81.63	0.360
Correct medication	90.20	84.71	93.13	<0.05
Harmful drugs (i.e., antibiotics, glucose drugs)	6.12	10.59	3.75	<0.05
Referral (%)	27.12	39.09	20.41	<0.01

VC, Village Clinic; THC, Township Health Center; PPC, Practicing Physician Certificate.

Data source: Authors' survey.

**Table 3:** Main outcomes of interactions in clinical vignettes for angina cases.

**Main outcomes of interactions in vignettes**	**Total (*N* = 306)**	**VC (*n* = 110)**	**THC (*n* = 196)**	***P***
	**%/*n***	**%/*n***	**%/*n***	
**Process quality**				
Number of recommended questions and exams (out of 25)	7.42	5.56	8.45	<0.01
% of recommended questions and exams	29.66	22.25	33.82	<0.01
Number of essential questions and exams (out of 10)	4.37	3.08	5.10	<0.01
% of essential questions and exams	43.73	30.82	50.97	<0.01
**Diagnosis quality (%)**				
Gave any diagnosis	95.10	92.73	96.43	0.150
Correct diagnosis	20.62	14.71	23.81	0.067
**Quality of treatment (%)**				
Gave any treatment plan	99.67	100	99.49	0.453
Correct treatment	77.38	82.73	74.36	0.093
Partly correct treatment	78.69	86.36	74.36	<0.05
Gave any medication	60.13	53.64	63.78	0.082
Correct medication	39.67	23.73	47.20	<0.01
Correct medication (including proprietary Chinese medicine)	73.91	64.41	78.4	<0.05
Antibiotics	5.98	13.56	2.4	<0.01
Referral (%)	68.30	83.64	59.69	<0.01

VC, Village Clinic; THC, Township Health Center.

Data source: Authors' survey.

#### Diabetes cases

Table 2 reports the results of clinical vignettes for the diabetes cases. For the diabetes clinical vignettes, the number of recommended questions asked, or exams requested by providers, was significantly higher in the THCs than in the VCs (8 out of 29, or 26.9%; and 5 out of 29, or 16.7%; respectively; *p* < 0.01). The proportion of the THC providers who correctly diagnosed diabetes was significantly higher than the proportion of the VC providers (97.4 and 87.3%, respectively; *p* < 0.01). Similarly, there were significantly more THC providers than VC providers who accurately diagnosed type 2 diabetes (48.2 and 28.2%, respectively; *p* < 0.01). Moreover, a significantly larger proportion of providers from THCs gave correct treatment plans to patients than providers from VCs (46.5 vs. 24.8%, *p* < 0.01), and significantly more THC providers gave the correct medicine regimes (93.1%, *p* < 0.05) than VC providers (84.7%). Last, regarding the number of referrals, the VC providers made significantly more referrals (39%, *p* < 0.01) than the THC providers (20).

#### Angina cases

Table 3 presents specific outcomes from the angina clinical vignettes for the THC and VC providers. Providers from the THCs asked or requested significantly more of the 25 recommended questions and exams than the VC providers (33.8 and 22.3%, respectively; *p* < 0.01), as well as significantly more of the ten essential questions (51 and 30.8%, respectively, *p* < 0.01). There were no significant differences in the quality of diagnosis between the VC and THC providers. The proportion of the VC providers that gave a partly correct treatment plan was significantly greater than the proportion of the THC providers (86.4 and 74.4%, respectively; *p* < 0.05). Furthermore, the proportion of the providers that prescribed the correct medication (including proprietary Chinese medicine) was significantly higher in the THCs than in the VCs (78.4 and 64.4%, respectively; *p* < 0.05), while significantly more providers from the VC facilities gave antibiotics (13.6 and 2.4%, respectively; *p* < 0.01). Last, a significantly larger proportion of the VC providers made referrals for angina cases than the THC providers (83.6 and 49.7%, respectively; *p* < 0.01).

### Standardized patients

**Figure 1:**
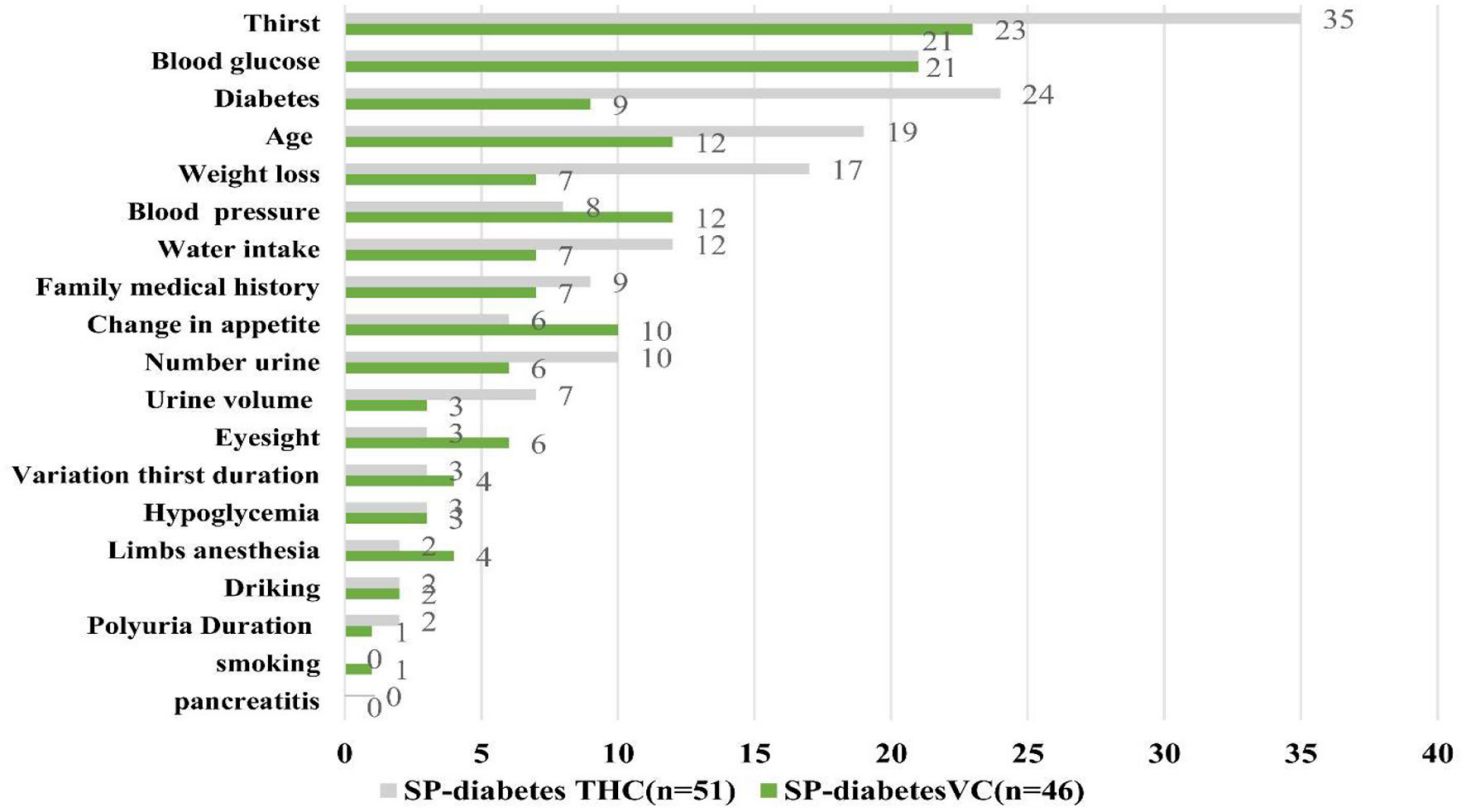
Adherence to clinical checklist, diabetes case.

**Figure 2:**
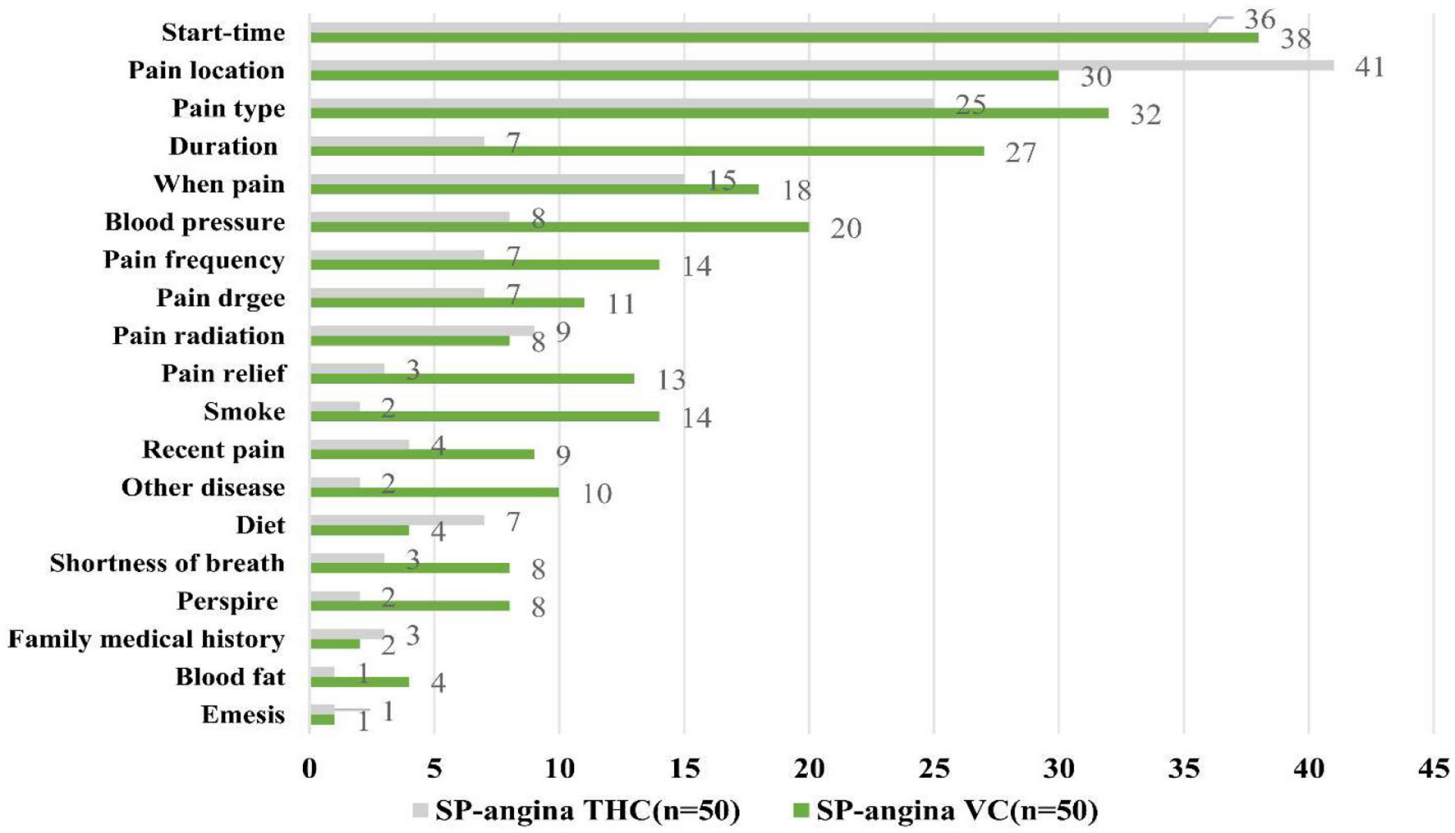
Adherence to clinical checklist, angina case.

Tables 4, 5 and Figures 1, 2 show the results of provider performance when interacting with SPs for diabetes and angina cases, respectively, as measured by diagnostic process quality, quality of the diagnosis given, and the quality of the treatment.

**Table 4:** Main outcomes of interactions with Standardized Patients (SPs) for diabetes.

**Main outcomes of interactions with SPs**	**Total (*N* = 97)**	**VC (*n* = 46)**	**THC (*n* = 51)**	***p***
	**%/*n***	**%/*n***	**%/*n***	
**Process quality**				
Number of recommended questions and exams (out of 29)	4.95	4.41	5.43	<0.05
% of recommended questions and exams	17.06	15.22	18.73	<0.05
Number of essential questions and exams (out of 9)	2.72	2.51	2.88	0.219
% of essential questions and exams	30.27	27.93	32.00	0.219
**Diagnosis quality (%)**				
Gave any diagnosis	94.85	95.65	94.12	0.733
Correct diagnosis (diabetes)	80.43	65.91	93.75	<0.01
Correct diagnosis (type 2 diabetes)	4.35	6.82	2.08	0.266
**Quality of treatment (%)**				
Gave any treatment plans	96.91	93.48	100.00	0.064
Partly correct treatment	87.50	80.00	93.75	0.052
Correct treatment	34.04	30.23	37.25	0.474
Gave advice on diet, lifestyle, and exercise	59.57	55.81	62.75	0.495
Instructed patient to monitor blood glucose regularly	54.26	44.19	62.75	0.072
Gave any medication	45.36	63.04	29.41	<0.01
Correct medication	50.00	41.38	66.67	0.112
Antibiotics	9.09	10.34	6.67	0.687
Harmful drugs (i.e., antibiotics, glucose drugs)	38.64	44.83	26.67	0.241
Referral (%)	17.53	21.74	13.73	0.300

VC, Village Clinic; THC, Township Health Center.

Data source: Authors' survey.

**Table 5:** Main outcomes of interactions with Standardized Patients (SPs) for angina.

**Main outcomes of interactions with SPs**	**Total (*N* = 100)**	**VC (*n* = 50)**	**THC (*n* = 50)**	***P***
	**%/*n***	**%/*n***	**%/*n***	
**Process quality**				
Number of recommended questions and exams (out of 25)	6.41	5.29	7.36	<0.01
% of recommended questions and exams	25.65	21.14	29.44	<0.01
Number of essential questions and exams (out of 10)	3.49	2.95	3.94	<0.01
% of essential questions and exams	34.89	29.52	39.40	<0.01
**Diagnosis quality (%)**				
Gave any diagnosis	94.00	94.00	94.00	1.000
Correct diagnosis	19.15	12.77	25.53	0.116
**Quality of treatment (%)**				
Gave any treatment plan	93.00	100.00	86.00	<0.01
Correct treatment	58.06	58.00	58.14	0.989
Partly correct treatment	64.52	66.00	62.79	0.747
Gave any medication	58.00	66.00	50.00	0.105
Correct medication,	15.52	9.09	24.00	0.120
Correct medication (including proprietary Chinese medicine)	43.10	27.27	64.00	<0.01
Antibiotics	25.86	30.30	20.00	0.375
Referral (%)	57.00	64.00	50.00	0.157

VC, Village Clinic; THC, Township Health Center.

Data source: Authors' survey.

#### Diabetes cases

Table 4 reports the results from the interactions with the SPs that presented symptoms of diabetes. Compared to the VC providers, the THC providers asked or requested a significantly higher number of questions or exams for the SPs displaying signs of diabetes (5 out of 29, or 18.7%; vs. 4 out of 29, or 15.2%; *p* < 0.05). Notably, the THC and VC providers diagnosed diabetes at similar rates (94.1 and 95.7%, respectively; *p* = 0.733), although the THC providers were significantly more likely to correctly diagnose diabetes (94 and 66%, respectively; *p* < 0.01). Furthermore, the VC providers were significantly more likely than the THC providers to prescribe any medication to SPs (63 and 29.4%, respectively; *p* < 0.01); however, the VC providers were less likely to give correct medicines for diabetes treatment than the THC providers (41.4 and 66.7%, respectively; *p* = 0.112).

#### Angina cases

Table 5 presents the results regarding the providers from the SP visits for angina cases. When comparing the SP visits between the THC and VC providers, results show that the THC providers asked and requested significantly more of the recommended questions and exams than the VC providers (29.4 and 21.1%, respectively; *p* < 0.01), as well as essential questions and exams (39.4 and 29.5%, respectively; *p* < 0.01). There were no statistically significant differences in quality of diagnosis between VC and THC providers, with 94% of providers in both facilities giving any diagnosis, and 12.8% of VC providers and 25.5% of THC providers providing correct diagnosis (*p* = 0.116). Regarding the quality of treatment, however, the VC providers were significantly more likely to give their patients treatment plans than the THC providers (100 and 86%, respectively; *p* < 0.01), although they may not always be correct: in fact, ~58% of both the VC and THC providers provided the correct treatment plans. Furthermore, if medication was prescribed to the SPs (50% in THCs and 66% in VCs), the THC providers were significantly more likely to provide the correct medication, including proprietary Chinese medicine, than the VC providers (64 and 27.3%, respectively; *p* < 0.01).

### The know-do gap

Tables 6, 7 report results on the know-do gap between clinical vignette surveys and SP visits for diabetes and angina cases, respectively. The size of the gap is visually presented for diabetes in Figure 1 and angina in Figure 2.

**Table 6:** The know-do gap of diagnostic questions asked and examinations performed, quality of diagnosis, and quality of treatment given for diabetes cases in Village Clinics (VCs) and Township Health Centers (THCs).

	**Total (*****N*** = **94)**				**VC (*****n*** = **46)**				**THC (*****n*** = **48)**			
	**SP**	**Vignette**	**Gaps**	***P***	**SP**	**Vignette**	**Gaps**	***P***	**SP**	**Vignette**	**Gaps**	***P***
	**%/*n***	**%/*n***	**%/*n***		**%/*n***	**%/*n***	**%/*n***		**%/*n***	**%/*n***	**%/*n***	
	**(1)**	**(2)**	**(1) – (2) = (3)**	**(4)**	**(5)**	**(6)**	**(5) - (6) = (7)**	**(8)**	**(9)**	**(10)**	**(9) - (10) = (11)**	**(12)**
**Quality of diagnosis process**												
Number of recommended questions and exams (out of 29)	4.89	6.01	−1.12	<0.01	4.41	5.09	−0.68	0.204	5.35	6.90	−1.55	<0.05
% of recommended questions and exams	16.87	20.73	−3.85	<0.01	15.22	17.54	−2.32	0.204	18.46	23.78	−5.32	<0.05
Number of essential questions and exams (out of 9)	2.44	3.76	−1.32	<0.01	2.02	3.22	−1.20	<0.01	2.83	4.27	−1.44	<0.01
% of essential questions and exams	27.07	41.73	−14.66	<0.01	22.46	35.75	−13.29	<0.01	31.48	39.47	−15.97	<0.01
**Quality of diagnosis (%)**												
Gave any diagnosis	95.74	98.94	−3.19	0.174	95.65	100	−4.35	0.153	95.83	97.92	−2.08	0.557
Correct diagnosis (diabetes)	80.00	94.62	−14.62	<0.01	65.91	91.30	−25.40	<0.01	93.48	97.87	−4.39	0.296
Correct diagnosis (type 2 diabetes)	4.44	30.11	−25.66	<0.01	6.82	19.57	−12.75	0.075	2.17	40.43	−38.25	<0.01
**Quality of treatment (%)**												
Gave any treatment plan	96.81	98.94	−2.13	0.312	93.48	100	−6.52	0.078	100	97.92	2.08	0.315
Correct treatment	34.07	34.42	−0.34	0.961	30.23	26.09	4.15	0.664	37.50	42.55	−5.05	0.615
Partly correct treatment	83.52	91.40	−7.88	0.106	74.42	86.96	−12.54	0.133	91.67	95.74	−4.08	0.414
Gave advice on diet, lifestyle, and exercise	60.44	60.22	0.22	0.975	55.81	50.00	5.81	0.583	64.58	70.21	−5.63	0.558
Instructed patient to monitor blood glucose regularly	54.95	55.91	−0.97	0.895	44.19	47.83	−3.64	0.731	64.58	63.83	0.75	0.939
Gave any medication	44.68	81.91	−37.23	<0.01	63.04	78.26	−15.22	0.109	27.08	85.42	−58.33	<0.01
Correct medication	52.38	94.81	−42.42	<0.01	41.38	94.44	−53.07	<0.01	76.92	95.12	−18.20	<0.05
Harmful drugs (i.e., antibiotics, glucose drugs)	38.1	2.60	35.50	<0.01	44.83	2.78	42.05	<0.01	23.08	2.44	20.64	<0.05
Referral (%)	18.09	27.66	−9.57	0.118	21.74	36.96	−15.22	0.109	14.58	18.75	−4.17	0.584

SP, Standardized Patient.

Data source: Authors' survey.

**Table 7:** The know-do gap of diagnostic questions asked, and diagnosis and treatment given for angina cases in Village Clinics (VCs) and Township Health Centers (THCs).

	**Total (*****N*** = **94)**				**VC (*****n*** = **46)**				**THC (*****n*** = **48)**			
	**SP**	**Vignette**	**Gaps**	***P***	**SP**	**Vignette**	**Gaps**	***P***	**SP**	**Vignette**	**Gaps**	***P***
	***%/n***	***%/n***			***%/n***	***%/n***			***%/n***	***%/n***		
	**(1)**	**(2)**	**(1) – (2) = (3)**	**(4)**	**(5)**	**(6)**	**(5) - (6) = (7)**	**(8)**	**(9)**	**(10)**	**(9) - (10) = (11)**	**(12)**
**Quality of diagnosis process**												
Number of recommended questions and exams (out of 25)	6.04	6.64	−0.60	0.218	4.74	5.35	−0.61	0.289	7.29	7.88	−0.59	0.395
% recommended questions and exams	24.17	26.55	−2.38	0.218	18.96	21.39	−2.43	0.289	29.17	31.50	−2.33	0.395
Number of essential questions and exams (out of 10)	3.29	3.97	−0.68	<0.05	2.63	3.02	−0.39	0.278	3.92	4.88	−0.96	<0.05
% essential questions and exams	32.87	39.68	−6.81	<0.05	26.30	30.22	−3.91	0.278	39.17	48.75	−9.58	<0.05
**Quality of diagnosis (%)**												
Gave any diagnosis	93.62	94.68	−1.06	0.756	93.48	95.65	−2.17	0.646	93.75	93.75	0.00	1.000
Correct diagnosis	20.45	16.85	3.60	0.539	13.95	15.91	−1.96	0.798	26.67	17.78	8.89	0.310
**Quality of treatment (%)**												
Gave any treatment plan	93.62	100.00	−6.38	<0.05	100	100.	0.00	1.000	87.50	100	−12.50	<0.05
Correct treatment	60.23	73.40	−13.18	0.059	63.04	82.61	−19.57	<0.05	57.14	64.58	−7.44	0.470
Partly correct treatment	65.91	76.60	−10.69	0.111	69.57	89.13	−19.57	<0.05	61.90	64.58	−2.68	0.792
Gave any medication	56.38	57.45	−1.06	0.883	63.04	50.00	13.04	0.207	50.00	64.58	−14.58	0.149
Correct medication	15.09	37.04	−21.94	<0.01	10.34	26.09	−15.74	0.136	20.83	45.16	−24.33	0.060
Antibiotics	24.53	11.11	13.42	0.069	27.59	13.04	14.54	0.202	20.83	9.68	11.16	0.245
Referral (%)	59.57	67.02	−7.45	0.290	67.39	86.96	−19.57	<0.05	52.08	47.92	4.17	0.683

SP, Standardized Patient.

Data source: Authors' survey.

#### Diabetes cases

**Figure 3:**
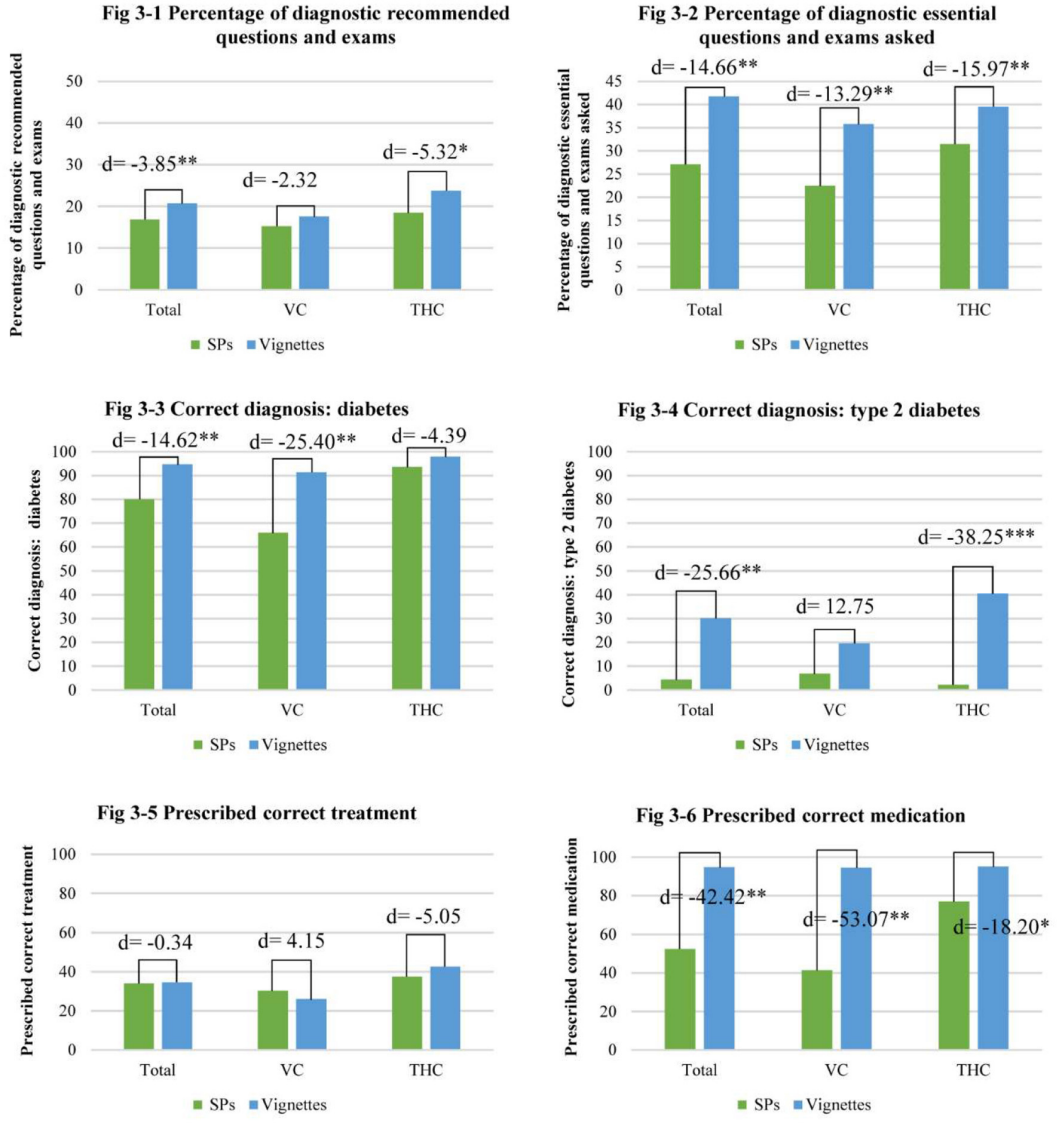
The know-do gap of diagnostic questions asked and diagnosis and treatment given for diabetes cases (*p < 0.05, **p < 0.01, ***p < 0.001).

Table 6 shows the know-do gap for diabetes cases. Overall, there were significant differences between provider knowledge and actions in the SP visits (where provider “do” is measured) and the clinical vignettes (where provider “know” is measured). Although the providers asked or requested 20.7% of the recommended diagnostic questions and exams in the clinical vignettes, the same providers asked and requested 16.9% of the recommended diagnostic questions and exams during the SP visits (*p* < 0.01). Regarding essential questions and exams for patients with diabetes, 41.7% of the providers asked the essential questions in vignettes, only 27.1% asked the SPs (*p* < 0.01). The proportion of cases that correctly diagnosed diabetes was 14.6% points higher in the vignettes than in the SP visits (*p* < 0.01), and the proportion of correctly diagnosed type 2 diabetes cases was 35.7% points higher in the vignettes than in the SP visits (*p* < 0.01). Notably, there are prominent know-do gaps regarding the medication prescribed during the clinical vignettes and SP visits. Overall, the providers gave the correct medication more often during the clinical vignette surveys than during the SP visits (Table 6; Figure 3): 94.8% of providers gave the correct medication during vignettes, while 52.4% of providers gave the correct medication to SPs, resulting in a know-do gap of 42.4% points (*p* < 0.01). Moreover, significantly more providers gave harmful drugs (i.e., antibiotics, sugar drugs, etc.) to SPs than they gave during vignettes (38.1 and 2.6%, respectively), resulting in a know-do gap of 35.5 percent points (*p* < 0.01).

For the VCs, specifically, there were significant know-do gaps across the quality of diagnosis process, quality of diagnosis, and quality of treatment. The VC providers asked significantly more essential questions to the mock patients in the vignettes (3.2 out of 9 questions, or 35.8%) than to the SPs (2 out of 9 questions, or 22.5%), resulting in a know-do gap of 1.2 questions, or 13.3% points (*p* < 0.01). There was also a significant know-do gap of 25.4% points (*p* < 0.01) between the proportion of correct diabetes diagnoses during the vignettes (91.3%) and the SP visits (65.9%). Last, there were significant know-do gaps between correct and incorrect prescription of medications for diabetes cases (*p* < 0.01). Specifically, the proportion of correct medication prescriptions in the vignettes was 53.1% points higher than the proportion of the VC providers that correctly prescribed medication to the SPs. Additionally, there was a significant gap of 42.1% points (*p* < 0.01) between the VC providers that gave harmful drugs to patients, with 2.8% of VC providers giving harmful drugs during the vignettes, and 44.8% of the VC providers prescribing harmful drugs to SPs.

For the THC providers, there were also significant know-do gaps across the quality of diagnosis process, the quality of the diagnosis, and the quality of treatment. Out of the 29 recommended questions and exams for diabetes cases, there was a significant know-do gap of−1.6 questions (or 5.3% points; *p* < 0.05) between THC providers in the vignettes (6.9 out of 29 questions, or 23.8%) and the THC providers in the SP visits (5.4 out of 29 questions, or 18.5%). Regarding the essential questions and exams for diabetes, there was a significant know-do gap of 1.4 questions (or −16%; *p* < 0.01) between the THC providers in the vignettes (4.3 questions, 39.5%) and the THC providers during the SP visits (2.8 questions, or 31.5%). There was also a significant know-do gap of 38.3% points (*p* < 0.01) between the proportion of the THC providers that gave a correct diagnosis of type 2 diabetes during the vignettes and SP visits (40.4 and 2.17%, respectively). Finally, for the THC providers, there were significant gaps between what the providers knew and did regarding giving medication during the vignettes and SP visits. Specifically, there was a significant gap of 58.3% points (*p* < 0.01) between the THC providers that gave any medication to patients during vignettes (85.4%) and during the SP visits (27.1%). There were also significant gaps between correct and harmful medications prescribed across the vignette and SP visits, resulting in a know-do gap of 18.2% points between the 95.1% of that THC providers that prescribed correct medication during vignettes, and the 76.9% of the providers that prescribed correct medication during SP visits (*p* < 0.05). Moreover, 2.4% of the THC providers in the vignettes prescribed harmful drugs to patients, and 23.1% of the THC providers gave harmful drugs to patients during the SP visits, indicating a significant know-do gap of 20.6% points (*p* < 0.05).

#### Angina cases

**Figure 4:**
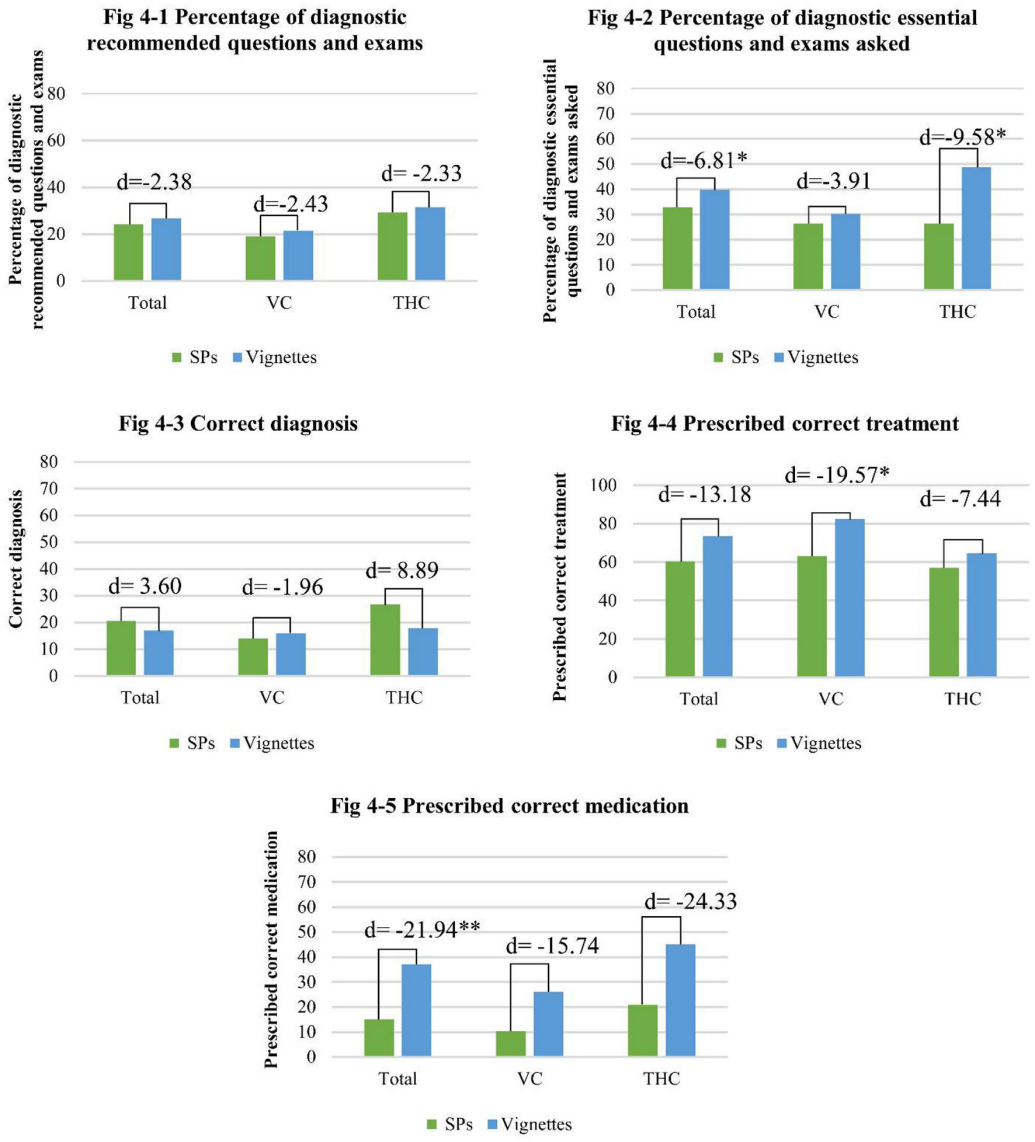
The know-do gap of diagnostic questions asked and diagnosis and treatment given for angina cases (*p < 0.05, **p < 0.01).

For angina cases, overall, there were only significant know-do gaps in diagnosing processes and the quality of treatment, as shown in Table 7. Across all facilities, the providers asked or requested 39.7% of the 10 essential questions and exams in the clinical vignettes while they asked or requested 32.9% of the essential questions and exams when treating the SPs (know-do gap of −6.8% points; *p* < 0.05). Furthermore, there were significant differences in the quality of treatment between the providers in the vignettes and SP visits. Specifically, the proportion of the providers that gave the correct treatment plan was 13.2% points higher in the clinical vignettes than in the interactions with the SPs (see Figure 4). Additionally, the know-do gap for the correct prescription of medication was 22% points (*p* < 0.01), with 15.1% of the providers prescribing the correct medication to the SPs, and 37.0% of the providers prescribing the correct medication in the clinical vignettes.

For the providers working in VCs, there were significant know-do gaps in the quality of treatment. Compared to the 82.6% of the providers that gave the correct treatment plan during the vignettes, only 63.0% gave the correct treatment plan to the SPs (know-do gap of 19.6% points, *p* < 0.05). There were no significant know-do gaps regarding the process of diagnoses, the quality of the diagnosis, or prescription of medications among VC providers. However, VC providers were significantly more likely to refer patients to other health care providers in the vignettes (87%) than in the SP visits (67.4%), indicating a know-do gap of 19.57% points (*p* < 0.05).

The final part of Table 7 shows the know-do gaps among the THC providers in angina cases. There were significant know-do gaps in the diagnosis process, and the quality of treatment, however, there were no significant gaps across the quality of diagnosis, correct treatment plans, medication prescriptions, or referrals. There was a significant difference between the number of essential questions and exams asked or requested during the vignettes and SP visits. In the vignettes, the providers asked 4.9 out of 10 essential questions or exams (48.8%), and during the SP visits, providers asked 3.9 out of 10 (39.2%) questions, resulting in a know-do gap of 1 question or 9.6% points (*p* < 0.05). There was also a significant know-do gap of 12.5% points between the providers in the vignettes and SP visits giving any treatment plan to the patients, with 100% of the providers giving any treatment plan in the vignettes, and 87.5% of the providers giving any treatment plan to the SPs (*p* < 0.05).

## Discussion

Chronic NCDs, such as diabetes and angina, have become an increasingly severe public health problem in China, especially in its rural areas. Previous research suggests that the high prevalence of NCDs may be due to the poor quality of health care in rural China, which ultimately may stem from the know-do gap: the chasm between medical knowledge and medical practice that results in poor evaluations, misdiagnoses, and treatments. To better understand the know-do gap and its relation to poor health care in rural China, this study uses clinical vignette and SP methods in primary health care centers, specifically VCs and THCs, in rural Sichuan to identify gaps between provider knowledge and actions. Overall, this study found that VC and THC providers demonstrate low levels of medical knowledge, with less than half of providers correctly diagnosing type 2 diabetes, and less than a quarter of providers able to correctly diagnose angina. Provider performance was similarly low, with <5% of providers accurately diagnosing type 2 diabetes, and <20% of providers correctly diagnosing angina. The low rates of accurate diagnosis are consistent with other studies that find high rates of misdiagnosis among primary care providers in rural China (11, 12). Overall, providers offer more accurate diagnoses and treatment plans when knowingly being assessed than when treating incognito SPs, thereby indicating the know-do gap, especially low-quality patient care as one of the primary constraints on NCD diagnosis and treatment in rural China. However, we should not ignore the importance of clinical knowledge training to improve the quality of clinical care.

In terms of provider knowledge, providers in rural China's health care centers demonstrate generally low levels of knowledge in regard to the diagnosis and treatment of NCDs. Based on clinical medical guidelines, the providers should complete a set of recommended and essential diagnostic questions and examinations, regardless of the patient's physical condition at the time of presentation. A patient with chest pain, for example, should be asked about the location of the pain and then referred to a facility that has an electrocardiogram (EKG). In clinical vignettes, the providers asked less than a third of the recommended questions for both diabetes and angina diagnoses, and less than half of the essential questions the providers should ask patients for the same cases. After asking such few questions, the providers in both the THCs and VCs made few correct diagnoses, correctly diagnosing type 2 diabetes only 40% of the time, and angina only 20% of the time. These low rates of correct diagnosis are not uncommon among rural Chinese health care providers, as have been documented in studies (11, 12, 18).

Low knowledge was also demonstrated in our sample when the providers were asked to give treatment plans during the clinical vignettes: <40% of the diabetes cases and 77% of the angina cases were properly treated, indicative of low treatment knowledge. Despite there being a higher proportion of properly managed angina cases than diabetes cases, the correct medication for angina was prescribed in only 40% of vignette surveys. Furthermore, our findings also indicate that, in general, provider knowledge is significantly lower in VCs than in THCs, thus putting patients in VCs at greater risk of misdiagnosis and mistreatment. VCs across rural China consistently report low levels of provider knowledge for angina, as one study across several provinces indicated that the VC providers were limited in diagnosing unstable angina (16, 18). Diabetes and angina are the most common NCDs that appear in patients in the THCs and VCs, but if the knowledge of the provider regarding diagnosing and treating NCDs remains low, rural community members and patients will continue to suffer from misdiagnosed, undiagnosed, mistreated, and untreated NCDs.

In addition to finding low levels of provider knowledge, results from this study indicate that, in practice, the providers demonstrate even lower levels of applied knowledge when diagnosing and treating patients. During incognito SP visits, the proportions of the rural providers that completed all of the recommended questions and examination items for diabetes and angina were 17 and 26%, respectively, which are similar to results from a study conducted in central and western rural areas in China in 2015 (11). Other research on the quality of diagnosis and treatment has shown that higher quality initial consultations are associated with a higher likelihood that the providers will make the correct diagnoses and prescribe better treatment plans (11). In other words, the more detailed the consultation, the lower the probability of misdiagnosis and missed diagnosis.

Looking at provider diagnosis and treatment, we see that following low quality consultations, the rates of correct type 2 diabetes and angina diagnoses are extremely low (4 and 19%, respectively), and the proportion of providers giving correct medication for both diseases is similarly low (50 and 15%, respectively). Another phenomenon we see is inaccurate prescription of medications. In nearly 40% of the diabetes cases, the providers gave the SPs harmful drugs, and in a quarter of angina cases the SPs were given unnecessary antibiotics. Similar to the results from the vignettes, we find that, overall, the providers in the THCs perform significantly better than their counterparts in the VCs. However, the consistent lack of knowledge in practice is common across all rural health facilities in this study.

Consequently, there is a large know-do gap in the diagnosis and treatment of NCDs among rural providers. We see the largest know-do gaps in the correct diagnosis of type 2 diabetes (25.7%); the prescription of any, correct, and incorrect medication for diabetes (37.2, 42.4, and 35.5%, respectively) and the prescription of correct medication for angina (21.9%). Moreover, a large know-do gap occurs in diabetes cases, where nearly 95% of the patients were correctly diagnosed in the clinical vignettes, only 80% of the SPs were correctly diagnosed. Moreover, the gap in the correct prescription of diabetes drugs was 43%, while the proportion of the correct treatment was only 34%. As early treatment of diabetes focuses on adjusting lifestyle habits, 60% of the providers proposed suggestions on diet, lifestyle, and physical exercise, and required patients to monitor blood glucose regularly. Early detection is critical because long-term undiagnosed chronic diseases can have a number of negative effects, such as a higher risk of diabetes-related complications, as well as increased use of health services and other associated costs (5). There is a smaller “know-do” gap in the diagnostic process quality, which indicates that the sample providers were unable to effectively identify the condition, symptoms, and medical history of a patient during consultations. Ultimately, this know-do gap seriously affects the rest of the patient's diagnosis and treatment, and ultimately their health and risk of death (11, 16, 32).

Additionally, the gap in prescribing antibiotics for angina cases was 13.4%, meaning that providers knew not to prescribe antibiotics (in clinical vignettes), but the proportion of providers prescribing antibiotics increased by 13% points in “actual” clinical diagnosis and treatment with SPs. “Provider-induced demand,” whereby a provider forgoes cheaper alternatives to recommend a more expensive treatment or exam to generate greater profit, may be partly responsible for the high rate of prescription of unnecessary antibiotic medications (16). Another possible reason for this phenomenon may be the physicians being unsure or unable to ascertain whether there is an infection, and thus preemptively prescribing antibiotics to prevent the infection or treat an illness in case there is one (16, 33, 34). Understanding the causes of this apparent know-do gap is critical to reducing preventable death among patients with angina and requires more future research.

THCs and VCs are the primary clinical medical institutions available for rural residents of China, but at present, the quality of these rural medical facilities overall is low. The quality is so low, in fact, that it can repel rural patients from seeking care at THCs and VCs and lead them to seek help from higher level medical institutions. Increasing the quality of care provided general practitioners in lower-tier health centers is one method for improving diagnosis and treatment at THCs and VCs.

Clinical knowledge is one important factor affecting the quality of providers' clinical care, but it could not be ignored that there are other factors, such as available equipment, patient condition, environmental condition, and personal factors of providers.

Based on these findings, policymakers should increase the number of opportunities for providers to attend training, further their education, and continuously improve the level of their professional medical knowledge. However, training programs alone are insufficient, and the government should increase spending, for incomes of rural THC and VC providers as a way to incentivize better job performance. This use of better incentives have be verified (35), however, when enhancing the quality of clinical care of providers, say by principles of 3Es, where the three Es stand for Education, Emolument and Empowerment, respectively (36, 37) and introducing financial incentives, special attention should be given to the ethics of such reform, such as separating incentives between hospitals and providers (38). This adjustment of incentives, accompanied by greater supervision and management, on top of training and educational programs, can provide the necessary conditions to improve the ability of rural providers to correctly diagnose and treat common NCDs, thus narrowing the “know-do” gap, and reducing the overall pressure on the Chinese medical system.

## Limitations

There are several limitations to our study. First, we evaluate the management of two diseases: diabetes and angina. These two diseases were chosen for their appropriateness to the SP methodology, as symptoms are easy to hide and unlikely to result in invasive tests. These diseases also have the benefit of being diagnostically simple. As a result, we do not know how physicians may deal with more complicated communicable and non-communicable diseases. However, given that the SP cases were designed to be relatively straightforward to diagnose, we suspect that quality of care would decrease with more complicated diseases, whether they be communicable or non-communicable. Second, the SPs did not complete invasive tests, and it could be argued that the completion of these tests may have led to different treatment paths for real patients. We can therefore evaluate the treatment path only to the point that an invasive test was ordered. Finally, although SPs underwent intensive training aimed at standardizing their presentation of the disease case, there may, nevertheless, remain differences across the SPs. Any differences between the SPs, however, are unlikely to significantly affect results as the SPs were randomly assigned to providers.

## Conclusions

Ultimately, the findings from this study suggest that the know-do gap between provider knowledge and performance is significant and large among the primary health care centers in rural China. Moreover, this paper provides evidence of significantly low levels of medical knowledge among the providers from rural China's VCs and THCs that encounter patients with diabetes and angina. Coupled with low levels of provider knowledge, we identified even lower levels of quality patient care in interactions with the SPs, as the providers performed worse across diagnostic consultations, quality of diagnosis, and quality of treatment when treating the SPs than when acting in the clinical vignettes. The difference between the results from the clinical vignettes and SP visits thus indicates that it is a low quality of patient care, over low levels of provider knowledge, that is the primary constraint on the quality of NCD diagnosis and treatment in rural China.

## Data availability statement

The raw data supporting the conclusions of this article will be made available by the authors, without undue reservation.

## Ethics statement

The studies involving human participants were reviewed and approved by the Institutional Review Board (IRB) of Sichuan University, China (protocol number: K2019021). The patients/participants provided their written informed consent to participate in this study.

## Author contributions

SM, QW, and YW: original draft preparation. SM, YW, and HX: data analysis. SS, HZ, and SR: critical review of the contents and supervision. SM, QW, YW, HX, LL, RY, and YC: data collection. LP, MA, and S-ED: critical review of the manuscript. All authors read and approved the final manuscript.

## Funding

The authors were supported by the China Medical Board (Grant Number: CMB-18-297) and Sichuan Province Medical Association (Grant Number: S18009).

## Conflict of interest

The authors declare that the research was conducted in the absence of any commercial or financial relationships that could be construed as a potential conflict of interest.

## Publisher's note

All claims expressed in this article are solely those of the authors and do not necessarily represent those of their affiliated organizations, or those of the publisher, the editors and the reviewers. Any product that may be evaluated in this article, or claim that may be made by its manufacturer, is not guaranteed or endorsed by the publisher.

## Abbreviations

NCD: non-communicable diseases
IDF: International Diabetes Federation
PHC: Primary Health Care
THC: Township Health Center
VC: Village Clinic
SP: Standardized Patient
RMB: Renminbi
USD: United States Dollar
SD: Standard Deviation
PPC: Practicing Physician Certificate
EKG: electrocardiogram.
